# The association between preoperative edema and postoperative cognitive functioning and health-related quality of life in WHO grade I meningioma patients

**DOI:** 10.1007/s00701-019-03819-2

**Published:** 2019-02-13

**Authors:** David van Nieuwenhuizen, K. Mariam Slot, Martin Klein, Dagmar Verbaan, Esther Sanchez Aliaga, Jan J. Heimans, W. Peter Vandertop, Saskia M. Peerdeman, Jaap C. Reijneveld

**Affiliations:** 1 Department of Neurology, Amphia Hospital, Breda, The Netherlands; 2 Department of Neurology, VU University Medical Center, Amsterdam, The Netherlands; 3 Department of Neurosurgery, VU University Medical Center, Amsterdam, The Netherlands; 4 Brain Tumor Center Amsterdam, VU University Medical Center, Amsterdam University Medical Centers, PO Box 7057, 1007 MB Amsterdam, The Netherlands; 5 Department of Neurosurgery, Academic Medical Center, Amsterdam, The Netherlands; 6 Department of Neurology, Academic Medical Center, Amsterdam, The Netherlands; 7 Department of Medical Psychology, VU University Medical Center, Amsterdam, The Netherlands; 8 Department of Radiology, VU University Medical Center, Amsterdam, The Netherlands

**Keywords:** Cognition, Brain edema, Quality of life, Meningioma, Counseling, Surgery

## Abstract

**Background:**

Studies on the associations between preoperative cerebral edema, cognitive functioning, and health-related quality of life (HRQOL) in WHO grade I meningioma patients are virtually lacking. We studied the association between preoperative cerebral edema on postoperative cognitive functioning and HRQOL 6 months postoperatively in WHO grade I meningioma patients.

**Methods:**

Twenty-one consecutive WHO grade I meningioma patients, who underwent surgery, were matched individually for age, gender, and educational level to healthy controls. Tumor and edema volume were assessed on preoperative T1- and T2-weighted MRI images, respectively. At least 5 months postoperatively, functional status, cognitive functioning, and HRQOL, using a cognitive test battery and the Short-Form Health Survey (SF-36), were determined. The correlation between preoperative tumor and cerebral edema volume with postoperative cognitive functioning and HRQOL was investigated using Kendall’s tau coefficients.

**Results:**

Compared to healthy controls, patients had lower verbal memory capacity (*p* = .012), whereas HRQOL was similar to matched healthy controls. In all cognitive domains, postoperative functioning was much lower in patients with preoperative cerebral edema than in those without. There were significant correlations between preoperative cerebral edema and tumor volume and postoperative cognitive functioning. Preoperative cerebral edema and/or tumor volume were not associated with HRQOL.

**Conclusions:**

Our results suggest that WHO grade I meningioma patients with larger volumes of preoperative cerebral edema are more at risk of experiencing limitations in longer-term cognitive functioning than patients with no or less edema preoperatively. This is an important knowledge for neurologists and neurosurgeons treating patients with a meningioma. More studies regarding the effect of peritumoral edema on cognitive functioning in meningioma patients are necessary.

**Electronic supplementary material:**

The online version of this article (10.1007/s00701-019-03819-2) contains supplementary material, which is available to authorized users.

## Introduction

In patients with a primary intracranial tumor, cognitive deficits as well as epileptic seizures and their treatment might negatively affect health-related quality of life (HRQOL) [3, 11, 32]. Meningiomas are the most frequently reported intracranial tumors, accounting for approximately one-third of all central nervous system tumors [22]. Although the majority of meningiomas is benign, patients have long-term neurological problems that affect normal daily activities [30].

We have previously shown that many patients with suspected as well as with histologically proven World Health Organization (WHO) grade I meningiomas show subtle cognitive deficits that might be attributed to the tumor itself, the surgical treatment, or the occurrence of seizures and treatment with antiepileptic drugs (AED) [6, 31].

Frequently, meningiomas are an incidental finding in ancillary investigations following head trauma or a routine radiological checkup (e.g., “total body MRI”) [16, 24, 34, 35]. Proper selection for surgery of patients with an incidentally found meningioma is, however, hampered by lack of information concerning preoperative predictors for long-term cognitive functioning and HRQOL. For this group of patients, timing and choice of treatment remains a matter of debate.

Several studies have suggested that tumor-related edema, apart from the tumor itself and tumor-related seizures and their treatment, may have a negative impact on cognitive functioning [15, 28, 29, 34]. The reported incidence of edema in meningioma patients varies from 38 to 90% [13, 21, 23, 25].

The aim of this study is to determine whether there is an association between preoperative cerebral edema and postoperative cognitive functioning and HRQOL at least 5 months following surgery in patients with a WHO grade I meningioma.

## Methods and materials

### Patients

In this cross-sectional study, we included all consecutive patients treated surgically for a meningioma between November 1, 2005 and September 31, 2007, at the VU University Medical Center, which is a tertiary referral center for primary brain tumor patients in the Amsterdam metropolitan area with a total population of approximately 2.4 million people. Excluded were patients with atypical or malignant meningioma (WHO grades II or III) and patients who suffered from one of the following medical conditions, as these may interfere with normal cognitive functioning: other central nervous system (CNS) or non-CNS malignancy, cerebrovascular pathology, congenital CNS malformations, multiple sclerosis, Parkinson’s disease, organic psychosis, dementia, or schizophrenia. Furthermore, patients had to have sufficient proficiency of the Dutch language to be able to carry out the cognitive tests.

The treating physician invited the patients by letter to participate in the study. Of the 28 patients who met the inclusion criteria, 21 agreed to participate in the study; seven patients declined because they considered participation to be too burdensome. After written informed consent was obtained, an appointment was made for cognitive assessment.

Patient characteristics and preoperative symptoms are shown in Table 1.

**Table 1:** Patient characteristics and pre- and postoperative symptoms

Patient	Age	Gender	Pre-operative symptoms	Anatomical location of the tumor	Volume of tumor (cc)	Volume of edema (cc)	Volume of residual tumor (cc)	Symptoms 3 months after surgery	Time from surgery to assessment (months)	Postoperative radiotherapy
1	72	f	Cranial nerve palsy (n. V, VII, VIII)	Right cerebellopontine angle	37.0	1.2	21.2	Decrease in cranial nerve palsy	27	Yes
2	52	m	None	Right frontal	104.5	35.1	44.5	Gait disturbance caused by spinal cord tumor	25	Yes
3	41	f	Visual field deficit	Right sellar	1.7	.0	.0	None	19	No
4	52	f	None	Right temporal	47.3	2.7	.0	None	17	No
5	66	f	Seizures	Right frontoparietal	18.9	34.9	.0	None	15	No
6	42	f	Proptosis OD	Right frontotemporal	3.9	.0	1.5	Decrease in proptosis	13	No
7	45	f	Migraine	Right parietal	12.0	.0	.0	None	10	No
8	56	m	None	Right temporal	25.8	.5	4.6	None	10	No
9	35	f	Diplopia	Right cerbellopontine angle	6.1	.0	.0	None	6	No
10	51	m	Seizures	Left parietal	31.9	14.3	.0	Mild dysphasia	23	No
11	66	f	Seizures	Left parietal	17.7	36.5	.6	None	23	No
12	49	m	Hemiparesis right	Left parietal	31.9	12.1	.0	Seizures	20	No
13	64	m	Hemiparesis right, dysphasia	Left parietal	3.8	5.3	.0	None	14	No
14	47	f	Seizures	Left frontotemporal	31.1	43.8	.0	None	13	No
15	43	f	Seizures	Left frontotemporal	29.2	46.3	.0	None	12	No
16	59	v	None	Left frontal	4.8	39.1	.0	None	11	No
17	71	m	Visual loss OD	Sellar, midline, frontal	4.6	.0	.0	Increase in visual loss OD	23	No
18	57	m	Visual loss OD	Sellar midline	7.6	.0	.0	None	17	No
19	58	v	None	Olfactory groove, frontal, midline	35.5	25.1	1.0	None	15	No
20	50	v	Anosmia	Olfactory groove, frontal, midline	76.8	15.9	.0	Anosmia	10	No
21	46	v	Visual loss ODS	Olfactory groove, frontal. L > R	64.2	61.5	2.9	Decrease in visual loss ODS	5	No

*OD* oculus dexter, *ODS* oculi dexter et sinister

### Healthy controls

For cognitive function, patients were individually matched with healthy controls from the Maastricht Aging Study, which comprises a large cross-sectional study into the biomedical and psychological determinants of cognitive aging of 2000 healthy individuals aged 24 to 81 years [8]. For HRQOL, patient were individually matched with healthy controls from a national study that aimed to translate the Short-Form Health Survey (SF-36) for use among Dutch-speaking residents of The Netherlands [1]. Patients and healthy controls were individually matched with respect to gender, age, and educational level. Educational level was assessed by a Dutch scoring system consisting of an eight-point scale, ranging from unfinished primary education (level 1) to university level (level 8) [5].

The medical ethics committee of the VU University Medical Center approved the study protocol.

### Study measures

#### Functional status

Patients’ capacity to carry out life’s daily activities was assessed by means of the Barthel Activities of Daily Living Index [33]. Scores range from 0 to 20, with higher scores indicating higher levels of functional independence. The Karnofsky Performance Status (KPS) Scale was used as an overall indicator of patients’ level of physical functioning [9]. KPS scores range from 0 to 100, with higher scores indicating higher levels of functioning. Patients’ level of neurologic functioning was assessed by means of Neurological Functional Status Scale, with the score range from 1 to 4; higher scores represent higher levels of neurologic dysfunction [20].

#### Cognitive functioning

A wide range of cognitive functions was assessed by means of a standardized test battery (see Appendix 1 in the Supplementary Material). Individual cognitive test scores were converted into *z*-scores using the means and standard deviations (SDs) of the matched healthy controls as a reference. Subsequently, *z*-scores were transformed into the following six cognitive domains: executive functioning, working memory, verbal memory, attention, information processing speed, and psychomotor speed [11]. Dysfunction in cognitive functioning was defined as a *z*-score ≥ 1.5 SD below the mean of the healthy controls. The total time required to complete the battery was approximately 60 min. After completing the battery, patients also filled in a visual analogue scale, by which patients reported the amount of effort they had to invest to complete the cognitive testing battery.

#### Health-related quality of life

Self-reported HRQOL was measured with the Dutch translation of the SF-36 [1]. The SF-36 is composed of 36 items, organized into eight multi-item scales assessing physical functioning (PF), role limitation caused by physical health problems (RP), bodily pain (BP), general health (GH), vitality (VT), social functioning (SF), role limitation caused by emotional problems (RE), and mental health (MH). Raw scores are converted linearly to scales of 0 to 100, with higher scores representing better levels of functioning.

#### Magnetic resonance imaging

**Fig. 1:**
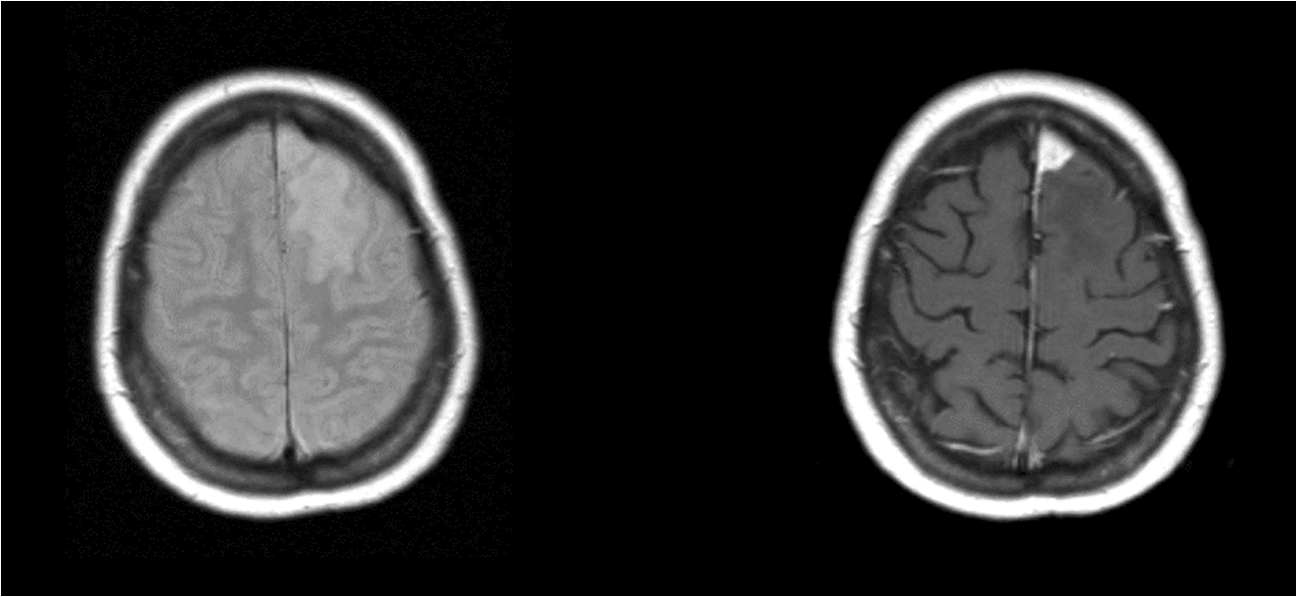
Magnetic resonance images. Meningioma with edema. Left image T2-weighted image; right image T1-weighted image with gadolinium

**Fig. 2:**
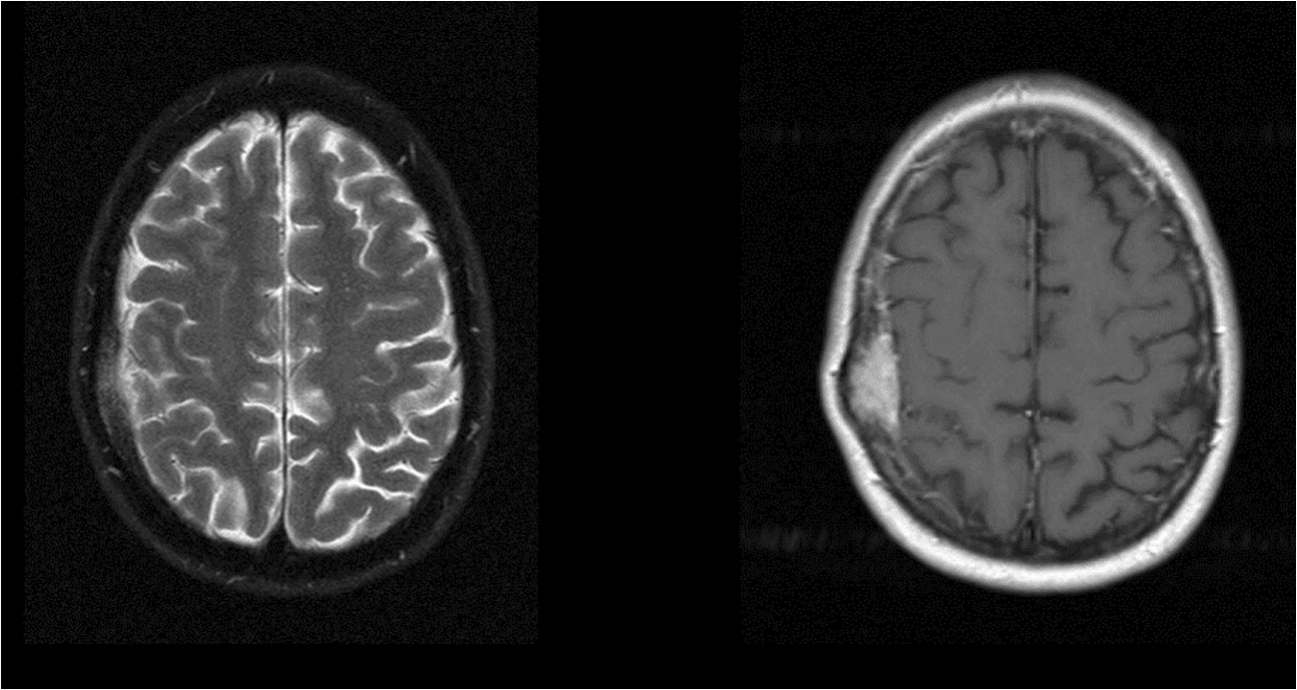
Magnetic resonance images. Meningioma without edema. Left image T2-weighted image; right image T1-weighted image with gadolinium

Magnetic resonance imaging (MRI) was performed prior to surgery for each patient. Scans were reviewed by a neuroradiologist (E.S.A.) who was not aware of the patient’s medical history. Data collection included tumor characteristics (tumor volume (cc), volume of edema (cc), total volume of edema and tumor (cc), and tumor localization). Edema was defined as T2-weighted hyperintensity adjacent to the tumor or present in the parenchyma adjacent to the surgical site. If there was no T2-weighted hyperintensity adjacent to the tumor or present in the parenchyma adjacent to the surgical site, we concluded that there was no edema present (example in Figs. 1 and 2). Volumetric measurement of tumor and T2-weighted hyperintensity was done using the BrainLAB iPlan® version 2.6 neuronavigation system (BrainLAB AG, Heimstetten, Germany) to seclude the meningioma.

### Statistical analysis

We determined a non-normal distribution because of the small study group. Mann-Whitney *U* tests were used to test for differences in cognitive functioning and HRQOL between patients and healthy controls. Furthermore, Kendall’s tau coefficients (two-tailed) were used to determine the associations between cognitive functioning or HRQOL and the following factors in both pre- and postoperative MRI scans: edema volume, tumor volume, and combined edema and tumor volume. In the statistical analyses performed using SPSS, version 24 for Windows, statistical significance was set at *p* < .05 (one-sided, because of the expected deleterious effects of having a meningioma and/or edema).

## Results

### Sociodemographic and clinical characteristics

As a result of the matching procedure, patients and healthy controls did not differ significantly in age, gender, and educational level (Table 2). Mean tumor volume in meningioma patients with edema was significantly larger than in meningioma patients without edema (37.4 vs 6.0 ml; *p* = .004).

**Table 2:** Sociodemographic and clinical characteristics of the study patients

	Meningioma patients (*n* = 21)	Healthy controls Cognitive functioning (*n* = 21)	*p* ^a^	Healthy controls HRQOL (*n* = 21)	*p* ^a^
	Mean (SD)	Mean (SD)		Mean (SD)	
Characteristics					
Mean age in years^d^	55.3 (10.6)	56.1 (10.4)	.82	55.9 (10.4)	.94
Gender, no. male (perc.)	7 (33.3)	9 (42.9)		6 (28.6)	
Educational level^d^	4.0 (1.8)	4.0 (1.8)	1.00	3.9 (1.9)	.75
Premorbid intelligence					
Dutch adult reading test (raw scores)	88.4 (14.3)	n/a		n/a	
Preoperative edema^c^					
Yes	15	n/a		n/a	
No	6	n/a		n/a	
Tumor characteristics^b^					
Tumor volume preoperative	28.4 (1.7–104.5^e^)	n/a		n/a	
Edema volume preoperative	17.8 (0.0–61.5^e^)	n/a		n/a	
Tumor volume 3 months postoperative	3.6 (0.0–44.5^e^)	n/a		n/a	
Edema volume 3 months postoperative	0.7 (0.0–6.7^e^)	n/a		n/a	
Tumor localization^c^					
Convexity, With edema Without edema	13 11 2	n/a		n/a	
Skull base, With edema Without edema	5 1 4	n/a		n/a	
Orbital, With edema Without edema	3 3 0	n/a		n/a	
Years since histological diagnosis	1.30 (0.52)	n/a		n/a	
Functional/performance status					
Karnofsky	100 (80–100)^f^	n/a		n/a	
Barthel	20 (8–20)^f^	n/a		n/a	

*HRQOL* health-related quality of life, *n/a* not applicable or not available

^a^Based on *t* test comparisons between patients and healthy controls

^b^Milliliters

^c^Number of patients

^d^Non-parametric *t* test

^e^(Minimum–maximum)

^f^Median (minimum–maximum)

### Cognitive functioning

Patients had a significantly lower verbal memory capacity (*p* = .012) compared to healthy controls. No statistically significant differences were found between patients and healthy controls in executive functioning, working memory, attention functioning, information processing speed and psychomotor speed. Patients with edema (*n* = 15) had significantly worse cognitive functioning than patients without edema (*n* = 6) in all six cognitive domains (Table 3).

**Table 3:** Cognitive functioning and health-related quality of life in meningioma patients with and without pre-operative peritumoral edema

	Meningioma patients with edema (*n* = 15)	Meningioma patients without edema (*n* = 6)	p*
Clinical characteristics			
Mean age in years	57.3 (8.9)	50.5 (13.8)	.118
Time of assessment since histological diagnosis (years)	1.3 (0.5)	1.2 (0.5)	.697
Tumor volume pre-operative	37.4 (26.9)	6.0 (3.6)	.004^a^
Tumor volume 3 months postoperative	5.0 (12.2)	0.3 (0.6)	.286
Percentage of symptomatic patient (#)	66.7% (10)	100% (6)	
Percentage of patients treated with radiotherapy after surgery (#)	13.3% (2)	0% (0)	
Domains of cognitive functioning^b^			
Executive functioning	− .6763 (1.2945)	1.0059 (0.5870)	.004^c^
Psychomotor speed	− .6544 (1.0200)	.4561 (0.3593)	.006^c^
Working memory	− .4349 (1.0145)	.7695 (0.6759)	.006^c^
Information processing	− .5683 (1.2087)	.8458 (0.5470)	.004^c^
Attention	− 1.0760 (2.0279)	.9396 (0.8111)	.006^c^
Verbal memory	− .3504 (.8069)	1.0908 (0.4257)	.023^c^
HRQOL SF36 Scales^d^			
Physical functioning	76.3 (25.4)	88.3 (23.8)	.14
Role physical	55.0 (45.5)	70.8 (45.9)	.36
Bodily pain	65.9 (26.9)	77.0 (29.0)	.34
General health	63.8 (17.7)	75.0 (20.9)	.18
Vitality	62.3 (16.8)	63.3 (26.4)	.67
Social functioning	73.3 (24.0)	85.4 (30.0)	.16
Role emotional	71.1 (41.5)	77.7 (40.4)	.75
Mental health	74.9 (16.7)	74.7 (28.4)	.88

Results are mean with (standard deviation). (#) number of patients

^a^Significantly different (*p* < .05) using Mann-Whitney *U* test (two-tailed)

^b^For the neuropsychological characteristics *z*-scores derived from the mean and SD of the healthy controls are displayed

^c^Significantly different (*p* < .05) from the Meningioma patients using Mann-Whitney *U* test (one-tailed)

^d^*HRQOL*, health-related quality of life; *SF36*, Short-Form Health Survey questionnaire; *M*, the mean value for each variable

### Health-related quality of life

Mann-Whitney *U* test (one-tailed) yielded no significant differences between patients and healthy controls in any of the eight multi-item scales assessing HRQOL (physical functioning *p* = .180, role limitations caused by physical health problems *p* = .479, bodily pain *p* = .237, general health *p* = .220, vitality *p* = .192, social functioning *p* = .448, role limitations caused by emotional problems *p* = .420 and mental health *p* = .391). The HRQOL of patients with edema did not differ from patients without edema in any of the eight SF-36 scales (see Table 3).

### Associations of tumor and edema volume with cognitive function and HRQOL

Correlational analyses showed significant associations between preoperative peritumoral edema volume and all cognitive domains postoperatively, except verbal memory and attention (Table 4). Considering preoperative tumor volume, analysis showed significant associations with postoperative executive functioning, psychomotor speed, and working memory. When preoperative edema and tumor volume were combined, significant associations were found for all cognitive domains, except for attentional functioning and verbal memory. Furthermore, post hoc analysis yielded significant positive associations between the extent of preoperative edema and preoperative tumor volume (*r =* .351; *p =* .030, Kendall’s tau, two-tailed).

**Table 4:** Correlations between cognitive domains, health-related quality of life and neuropathological variables

	Preoperative edema	Preoperative tumor volume	Combined preoperative tumor volume and edema
Domains of cognitive functioning			
Executive functioning	*r* = − .331*	*r* = − .357**	*r* = − .357*
Psychomotor speed	*r* = − .301*	*r* = − .324*	*r* = − .314*
Working memory	*r* = − .381**	*r* = − .305*	*r* = − .352*
Information processing	*r* = − .321*	*r* = − .219	*r* = − .305*
Attention	*r* = − .262	*r* = − .219	*r* = − .190
Verbal memory	*r* = − .173	*r* = − .152	*r* = − .124
HRQOL SF36 Scales			
Physical functioning	*r* = − .221	*r* = − .137	*r* = − .208
Role physical	*r* = − .288	*r* = − .216	*r* = − .306
Bodily pain	*r* = − .278	*r* = .051	*r* = − .132
General health	*r* = − .172	*r* = − .000	*r* = − .050
Vitality	*r* = − .041	*r* = .089	*r* = − .089
Social functioning	*r* = − .263	*r* = − .237	*r* = − .227
Role emotional	*r* = − .142	*r* = − .019	*r* = − .031
Mental health	*r* = − .088	*r* = − .010	*r* = − .040

Non-parametric correlation, Kendall’s tau *b* test. *r* means correlation coefficient

*HRQOL SF36* health-related quality of life Short Form 36

*Correlation is significant at the p < .05 level

**Correlation is significant at the *p* < .01 level

No significant associations were established for preoperative edema, preoperative tumor volume, or the combination of edema and tumor volume with HRQOL.

## Discussion

We assessed the association between preoperative cerebral edema and postoperative cognitive functioning and HRQOL after surgery for a WHO grade I meningioma and found that patients with preoperative edema had significantly worse cognitive functioning than meningioma patients without preoperative edema after at least 5 months’ follow-up. However, there was no difference in HRQOL between both patient groups 6 months after surgery.

Little is known regarding the effects of edema on cognitive functioning of meningioma patients. Steinvorth et al. evaluated cognitive outcome in patients with skull base meningiomas after fractioned stereotactic radiotherapy and only found a transient decline in memory functioning, 1 day after treatment. The authors concluded that this decline was most likely related to an increase in pre-existing peritumoral edema due to radiotherapy [28]. Tucha et al. showed that preoperative edema was not associated with cognitive functioning 4 to 9 months after surgery [29]. In our study, larger volumes of preoperative edema were significantly associated with lower postoperative cognitive functioning.

Peritumoral edema in meningioma patients is considered to be vasogenic. Possible causative factors for this edema, e.g., tumor volume, tumor location, vascular supply, venous obstruction, histology, vascular endothelial growth factor (VEGF) production, and interleukin-6 expression, have been studied, but the exact mechanism of development of cerebral edema in meningioma patients remains unclear [4, 17, 21, 36]. Due to the blood-brain-barrier disruption, fluid is transferred to the extracellular space leading to disturbances of synaptic functioning and signal conduction across axons and dendrites [28]. White matter is particularly vulnerable to vasogenic edema, which tends to extend along neighboring fiber tracks. Furthermore, edema might lead to mass effect and thus to further compression of surrounding brain, sometimes resulting in local ischemia. These mechanisms may lead to cognitive deficits.

Cognitive deficits in brain tumor patients might also be attributed to surgical treatment, to the occurrence of seizures, to the treatment with antiepileptic drugs, or to the tumor itself [6, 11, 12]. Studies describing the association between tumor volume and the extent of peritumoral edema are inconsistent [2, 7, 10, 27]. In a previous study, we have shown that the addition of radiotherapy after surgery does not seem to have significant early (median follow up 3.3 years) detrimental impact on an already impaired cognitive functioning in meningioma patients [31]. We found positive associations between preoperative tumor volume and postoperative cognitive functioning. Furthermore, post hoc analysis yielded positive associations between the extent of preoperative edema and preoperative tumor volume. Although the association between preoperative edema and cognitive functioning was stronger than the association between preoperative tumor volume and cognitive functioning, tumor volume is a possible confounding factor for the association found between edema and cognitive performance. Regarding the mechanical effects exerted by both the tumor and the surrounding edema, it is most likely that the combination of these volumes might have negatively affected neurocognitive outcome. A relatively recent study also showed larger tumor volumes to be associated with poorer neurocognitive outcomes [14]. Considering the fact that surgery led to a significant reduction in tumor and edema mass (Table 2), we do not expect postoperative residual tumor and edema to affect cognition. Although our study shows that preoperative peritumoral edema is associated with limitations in cognitive functioning after surgery, this preoperative peritumoral edema does not seem to affect HRQOL. A recent systematic review by Najafabadi et al. describes both worse and better HRQOL scores in meningioma patients compared with healthy controls. Based on the available results, they conclude that, in general, meningioma patients have a clinically relevant worse HRQOL than healthy controls [19]. In our study, HRQOL in treated meningioma patients was comparable to that of the general population. A correlation between preoperative edema and HRQOL was not found. As far as we know, the effect of peritumoral edema and tumor volume on HRQOL has not been studied in meningioma patients before. The relatively unaffected HRQOL in our study may be explained by the fact that patients might have adapted to their postsurgical functioning or that a patient’s understanding or perception of HRQOL has changed over time, the so-called response shift [26]. Furthermore, Waagemans et al. showed that HRQOL particularly is compromised in meningioma patients with major cognitive deficits and those using AEDs [32].

Some shortcomings of this study need to be addressed. The number of patients is too small for subgroup analyses. The effect of epilepsy burden (i.e., AED use and seizure frequency), localization and lateralization of the tumor, and treatment-related factors on cognitive functioning and HRQOL could therefore not be studied. Pre- and postoperative amount of tumor may also influence cognition. Multivariate analysis to correct for tumor volume could not be performed, because of the small amount of patients.

Furthermore, since preoperative cognitive assessment is lacking, we could not analyze whether surgery itself also influences cognition. The effects of surgery on cognition in meningioma patients are not clear. Markovic et al. suggest that postoperative complications are more frequent in meningioma patients with peritumoral edema, which indicates the possibility of cognitive decline after surgery [18]. However, Tucha et al. indicate postoperative improvements in attentional functions in meningioma patients [29].

Although our results suggest that meningioma patients with larger volumes of preoperative peritumoral edema are at risk of experiencing longer-term cognitive deficits, future research efforts should focus on underlying causes and the question whether (early) reduction of peritumoral edema results in an improvement of cognition. Perhaps peritumoral edema in otherwise asymptomatic patients should prompt earlier surgery, or early treatment with medication (e.g., steroids, angiogenesis inhibitors) in an effort to prevent, or ameliorate, longer-term cognitive functioning. To meet this aim, prospective studies with larger patient groups including pre- and postoperative assessment are needed.

## Conclusion

Our results suggest that meningioma patients with larger volumes of preoperative peritumoral edema may be at risk of experiencing limitations in longer-term cognitive functioning. This knowledge is useful for neurologists and neurosurgeons treating patients with a meningioma, but more studies regarding the effect of peritumoral edema on cognitive functioning in meningioma patients are necessary.

## Electronic supplementary material

ESM 1 (DOCX 17 kb)
