# Supplementary material for: The association between preoperative edema and postoperative cognitive functioning and health-related quality of life in WHO grade I meningioma patients
Source: Acta Neurochir (Wien). 2019 Feb 13;161(3):579–88. doi: 10.1007/s00701-019-03819-2 (PMC6407739; doi:10.1007/s00701-019-03819-2)
Supplement: Supplementary file 1 — (DOCX 17 kb) [file 701_2019_3819_MOESM1_ESM.docx]

Appendix 1: Description of the neuropsychological test battery

**Test domains**

Overall cognitive performance Intelligence

*The Dutch Adult Reading Test (DART)* [1]. The Dutch version of the New Adult Reading Test provides a measure of premorbid capacity based on verbal ability.

Perception

*Line Bisection Test* [2]. This test is a device for measuring unilateral neglect, which is usually a sequel of massive right hemisphere

lesions. Noticeable errors are most often made by patients with visual field defects who tend to underestimate the side of the line

opposite to the defective field. Outcome measures are horizontal and vertical deviations.

Memory

*Auditory Verbal Learning Test (AVLT)* [3]. This version of the Rey Auditory Verbal Learning Test calls for various aspects of verbal

learning and recall. Measures used for analysis are: memory performance on trial 1 as indicator of immediate recall, total recall after five trials, delayed recall and recognition after 20 min as indicators of memory consolidation into long-term memory, and a delta score as a measure of learning capacity.

*Working Memory Task (WMT)*[4]. This task is designed to measure the speed of memory processes. The underlying principle is that the extra time needed to complete a test in which there is a stepwise increase in the amount of information to be kept in memory, is a

measure of the ease at which information is processed in working memory. Capacity is measured by using the slope and intercept as a function of the number of letters to be kept in working memory.

Attention and executive function:

*Stroop Color-Word Test (SCWT)*[3]. This test is a selective attention task aiming at measuring interference susceptibility and consists of three subtasks with increasing task complexity.

*Categoric Word Fluency*[5]. This is a task requiring the generation of words from specific semantic categories (animals) within a

limited time.

*Concept Shifting Test (CST)* [6]. This test, which has two conditions of complexity, predominantly measures functions associated with executive function, especially visual scanning and conceptual tracking. The motor component of this task is measured by three

dummy conditions in which no neurocognitive capacity except for graphomotor speed is required.

Speed

*Letter–Digit Substitutiuon Test (LDST)* [7]. This test, measures psychomotor speed that is relatively unaffected by a decline in intellectual ability. Participants are required to replace the randomized letters with the appropriate digit indicated by the key, as quickly as possible.

1. Schmand B, Bakker D, Saan R, Louman J (1991) The dutch reading test for adults: a measure of premorbid intelligence level. Tijdschr Gerontol Geriatr 22(1):15–19
2. Schenkenberg T, Bradford DC, Ajax ET (1980) Line bisection and unilateral visual neglect in patients with neurologic impairment. Neurology 30:509–517
3. Lezak MD (2004) Neuropsychological assessment, 4th edn. Oxford University Press, New York
4. Sternberg S (1975) Memory scanning: new findings and current controversies. Q J Exp Psychol 27:1–32
5. Benton AL (1968) Differential behavioral effects in frontal lobe disease. Neuropsychologia 6:53–60
6. Houx PJ, Jolles J (1994) Vulnerability factors for age-related cognitive decline. In Isaacson RL, Jensen KF (Eds): The

vulnerable brain and environmental risks. Plenum Press, New York, pp 25–41

1. Jolles J, Houx PJ, van Boxtel MPJ, Ponds RWHM (1995) *Maastricht Aging Study: Determinants of cognitive aging*, Maastricht, The Netherlands: Neuropsych Publishers
